# P2Y12 antagonist attenuates eosinophilic inflammation and airway hyperresponsiveness in a mouse model of asthma

**DOI:** 10.1111/jcmm.12727

**Published:** 2015-11-27

**Authors:** Dong‐Hyeon Suh, Hoang Kim Tu Trinh, Jing‐Nan Liu, Le Duy Pham, Sang Myun Park, Hae‐Sim Park, Yoo Seob Shin

**Affiliations:** ^1^ Department of Allergy and Clinical ImmunologyAjou University School of MedicineSuwonKorea; ^2^ Department of PharmacologyAjou University School of MedicineSuwonKorea

**Keywords:** asthma, clopidogrel, eosinophil, leukotriene E4, P2Y12 receptor

## Abstract

Leukotriene E4 (LTE4) that plays a key role in airway inflammation is expressed on platelets and eosinophils. We investigated whether blocking of the P2Y12 receptor can suppress eosinophilic inflammation in a mouse model of asthma because platelets and eosinophils share this receptor to be activated. BALB/c mice were sensitized by intraperitoneal injection of ovalbumin (OVA), followed by OVA nebulization. On each challenge day, clopidogrel, a P2Y12 antagonist was administered 30 min. before each challenge. Forty‐eight hours after the last OVA challenge, mice were assessed for airway hyperresponsiveness (AHR), cell composition and cytokine levels, including chemokine ligand 5 (CCL5), in bronchoalveolar lavage (BAL) fluid. EOL cells were treated with LTE4, with or without clopidogrel treatment, and intracellular and extracellular eosinophil cationic protein (ECP) expressions were measured to find the inhibiting function of P2Y12 antagonist on eosinophilic activation. The levels of P2Y12 expression were increased markedly in the lung homogenates of OVA‐sensitized and ‐challenged mice after platelet depletion. Administration of clopidogrel decreased AHR and the number of airway inflammatory cells, including eosinophils, in BAL fluid following OVA challenge. These results were associated with decreased levels of Th2 cytokines and CCL5. Histological examination showed that inflammatory cells as well as mucus‐containing goblet cells were reduced in clopidogrel‐administered mice compared to vehicle‐treated mice. Clopidogrel inhibited extracellular ECP secretion after LTE4 stimulation in EOL‐1 cells. Clopidogrel could prevent development of AHR and airway inflammation in a mouse model of asthma. P2Y12 can be a novel therapeutic target to the suppression of eosinophils in asthma.

## Introduction

Eosinophils are key effector cells inducing airway inflammation and airway hyperresponsiveness (AHR) in allergic asthma. They contain abundant preformed and *de novo* inflammatory mediators, such as eosinophil cationic protein (ECP) and CysLTs 1, 2, 3. Human eosinophils not only produce and secrete CysLTs but also express both CysLT receptor 1 (CysLTR1) and CysLT receptor 2 (CysLTR2) on the cell surface as well as the outer membranes of their granules 4.

CysLTs, including LTC4, D4 and E4, are well‐known proinflammatory lipid mediators that are synthesized from membrane‐derived arachidonic acid *via* the 5‐lipoxygenase pathway and contribute to the pathogenesis of asthma by affecting leukocyte recruitment, enhancing activities of eosinophils 5. Among the 3 CysLTs, leukotriene E4 (LTE4) is the most stable and abundant form 6, and known to have great inflammatory effects on inducing bronchial constriction and enhancing inflammatory cell infiltration compared to LTC4 and LTD4 7, 8; however, LTE4 binds to CysLTR1 and CysLTR2 with low affinity 9, 10, 11, 12 compared to LTC4 and LTD4.

Using an *in silico* modeling, it is predicted that LTE4 could bind to purinergic receptors, such as P2Y12, and elicit calcium flux and phosphorylation of extracellular signal‐regulated kinase in the P2Y12 receptor overexpressing transfected cells 13. However, a more recent study suggested that LTE4 cannot activate signalling through P2Y12R 14, and G‐protein coupled receptor (GPR) 99 has been identified as a direct LTE4‐specific receptor because mice lacking CysLT1, CysLT2 and GPR 99 receptors lose the ability to respond to the CysLTs, including LTE4 15.

P2Y12 is an adenosine diphosphate chemoreceptor expressed on platelet membrane, and eosinophil granule membranes also express the P2Y12 receptor 4. A recent study reported an increased number of platelet‐adherent eosinophils in the upper and lower airways of patients with asthma 16. In addition, genetic polymorphisms of *P2RY12* can affect platelet and eosinophil activation in patients with asthma 17.

Clopidogrel, known as an antagonist to P2Y12, is a thienopyridine class antiplatelet agent that has been used to inhibit blood clots in the treatment of various cardiovascular diseases 16. In addition, the drug has been proposed to be a potential therapeutic for various inflammatory diseases due to its anti‐inflammatory and immunomodulatory effects observed in cardiovascular diseases 18. However, their anti‐inflammatory therapeutic effects in allergic asthma have not yet been completely understood.

In this study, we evaluated the blocking effect of the P2Y12 receptor antagonist on eosinophilic asthmatic inflammation because platelets and eosinophils share this receptor to be activated.

## Materials and methods

### Animals

Female 6‐week‐old BALB/c mice weighing 20 ± 2 g were purchased from Jackson Laboratory (Bar Harbor, ME, USA). All mice were housed under specific pathogen‐ and ovalbumin (OVA)‐free conditions and maintained on a 12‐hr light**–**dark cycle with food and water *ad libitum*. All animal experiments performed in this study were approved by the Institutional Animal Care and Use Committee of Ajou University (IACUC 2013‐0068).

### OVA sensitization, challenge and clopidogrel treatment

The experimental protocol for allergen sensitization and challenge was modified from previously described procedures 19. Briefly, BALB/c mice were sensitized intraperitoneally with 10 μg of OVA (Fisher Scientific, Pittsburgh, PA, USA) emulsified in 1 mg of alum (Imject Alum; Pierce, Rockford, IL, USA) on days 0 and 14. For the allergen challenge, mice were subjected to airway allergen challenges by exposure to 1% OVA aerosols for 20 min. on days 28, 29 and 30 with an ultrasonic nebulizer (NE‐U22; Omron, Kyoto, Japan). To treat P2Y12 antagonist, mice were administered by oral gavage with 30 mg/kg of clopidogrel (Sigma‐Aldrich, St. Louis, MO, USA) in PBS for 30 min. before each OVA challenge. PBS was given as a vehicle. Each experiment in our study was repeated 3 times with 5 mice per group.

### Airway resistance measurement, bronchoalveolar lavage and lung histology

Airway resistance to inhaled methacholine (MCh; Sigma‐Aldrich) was measured using the flexiVent System (SCIREQ, Montreal, QC, Canada) 48 hrs after the last OVA challenge, as previously described 20. Bronchoalveolar lavage (BAL) was performed immediately after assessing AHR *via* the tracheal cannula with 1 ml of Hank's balanced salt solution. Leukocytes were counted with a haemocytometer, and cell differentiation was performed on cytospin slides prepared with Wright‐Giemsa stain as previously described 21. Tissue sections were evaluated using ImageJ (National Institutes of Health, Bethesda, MD, USA). To detect inflammatory cells, sections were stained with haematoxylin and eosin, and mucus‐containing cells were stained with periodic acid‐Schiff (PAS). The number of inflammatory cells per μm^2^ of perivascular and peribronchial areas and the number of mucus‐containing cells per μm^2^ of basement membrane were determined.

### Enzyme‐linked immunesorbent assay

The levels of interleukin (IL)‐4, IL‐5, IL‐13, and interferon (IFN)‐γ in BAL fluid were measured using a sandwich ELISA (eBioscience, San Diego, CA, USA), and ECP levels in human eosinophil cells (EOL‐1) supernatants and CCL5 levels in BAL fluid were analysed by a quantitative ECP ELISA kit (Medical & Biological Labs, Nakaku Nagoya, Japan and R&D systems, Minneapolis, MN, USA, respectively).

### Platelet depletion

Platelets that express P2Y12 receptors were removed before measuring the P2Y12 level in lung homogenates. To remove platelets, sensitized mice intraperitoneally received 40 μg of rat monoclonal antibody against mouse CD42b (Emfret Analytics, Eibelstadt, Germany) 48 hrs before experiments.

### *In vitro* cell culture and Western blot

EOL‐1 cells were purchased from Sigma‐Aldrich. Cells (1 × 10^6^/ml) were seeded into each well of 6‐well plates, grown in RPMI‐1640 with 10% FBS and 1% penicillin/streptomycin under a 5% CO_2_ humidified atmosphere at 37°C. After reaching the appropriate cell confluence, the cells were pretreated with clopidogrel (10 μg/ml) or montelukast (10 μM) in serum‐free media for 30 min. Then, EOL‐1 cells were stimulated with 300 nM LTE4 for 30 min.

After stimulation, the cells were lysed in RIPA buffer containing protease inhibitor cocktail (Fisher Scientific). Total 30 μg of protein from each cell lysate were loaded onto a 15% SDS‐PAGE gel, subjected to electrophoresis, and transferred to Polyvinylidene fluoride (PVDF) membranes. The membranes were blocked by 5% skim milk in PBS containing 0.05% Tween‐20 and incubated with the appropriate secondary antibodies conjugated to horseradish peroxidase. Antibodies used against human target proteins were ECP (Santa Cruz Biotechnology, Dallas, TX, USA) and β‐actin (Santa Cruz Biotechnology).

### Immunohistochemistry

Immunohistochemistry (IHC) was performed using either the avidin‐biotin‐complex method or the immunofluorescence technique on 5‐μm‐thick paraffin sections. After deparaffinization, tissue sections were sequentially incubated with blocking buffer (0.05% PBS‐Tween 20 containing 5% bovine serum albumin and 10% normal horse serum) for 1 hr at room temperature (RT), and then incubated with rabbit anti‐ECP antibody (Santa Cruz Biotechnology), overnight at 4°C. The sections were incubated for 1 hr with goat biotinylated rabbit antibody (Vector, Burlingame, CA, USA) at RT, and then with the avidin‐biotinylated horseradish peroxidase complex for 1 hr at RT. Cell nuclei were counterstained with haematoxylin. For immunofluorescence labelling, tissue sections were blocked as previously described and incubated with rabbit anti‐P2Y12R antibody (Abcam, Cambridge, UK) overnight at 4°C in a humidified chamber. The sections were incubated with Alexa Fluor 488‐labeled donkey anti‐rabbit antibody (Life Technologies, Carlsbad, CA, USA) for 50 min. at 37°C. Sections were counterstained with 4′,6‐diamidino‐2‐phenylindole (DAPI; 0.5 μg/ml) for 2 min. at RT and mounted using Biomeda Mounting Media (Biomeda, Foster city, CA, USA).

### Immunocytochemistry

Bronchoalveolar lavage fluid cells (2× to 5 × 10^4^ cells/ml) were seeded onto Poly‐l‐lysine‐coated coverslips and allowed to attach to coverslips by incubation for 1 hr at 37°C. Bronchoalveolar lavage fluid cells were fixed with 4% paraformaldehyde for 10 min. at RT and consecutively incubated with blocking buffer for 1 hr. Primary antibodies against the P2Y12 receptor (Santa Cruz Biotechnology) and ECP (Santa Cruz Biotechnology) were added to the sections, followed by incubation in a humidified chamber for 1 hr at 37°C. Thereafter, the sections were incubated with appropriate secondary antibodies. The Alexa Fluor 594 donkey anti‐goat antibody (Life Technologies) and Alexa Fluor 488 donkey anti‐rabbit antibody (Life Technologies) were applied for 50 min. at 37°C. Finally, after nuclear stain with DAPI (0.5 μg/ml) for 5 min., Biomeda Mounting solution (Biomeda) was dropped on the glass slide, and the coverslips were inverted and placed onto glass microscope slides.

### Statistical analysis

The results are presented as the mean ± SEM. Comparisons among the study groups were performed by one‐way anova, followed by Tukey's *post hoc* test. All data were analysed using SPSS version 19.0 (SPSS Inc, Chicago, IL, USA), and a *P*‐value of less than 0.05 was considered statistically significant.

## Results

### The expression of P2Y12 is increased after OVA sensitization and challenge

**Figure 1:**
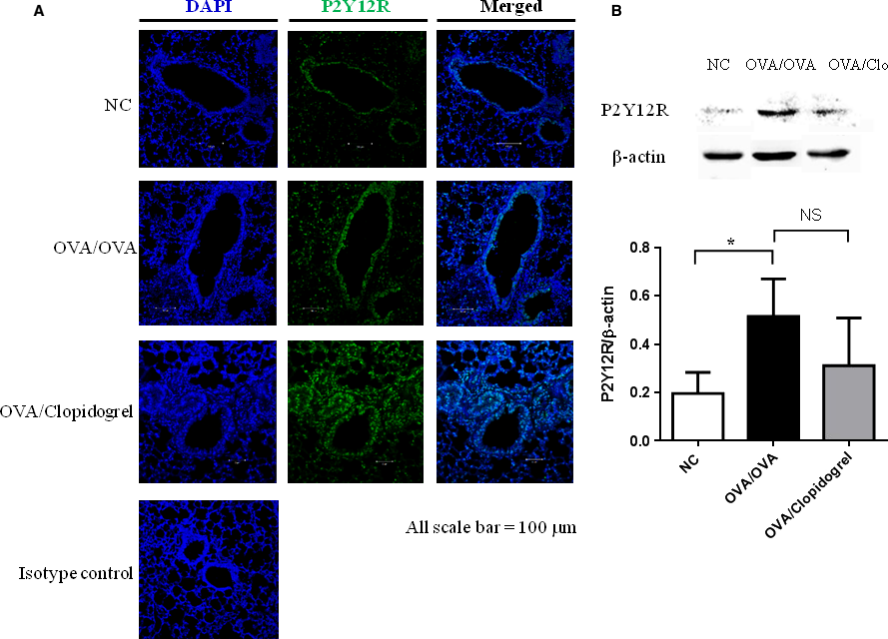
The expression of P2Y12 in lung tissue after platelet depletion. (**A**) P2Y12 protein levels in lung tissue. Immunofluorescence labelling and confocal microscopy were performed in lung sections using antibodies to the P2Y12 receptor counterstained with DAPI. (**B**) P2Y12 levels were determined by Western blot in lung tissue from OVA‐sensitized and saline‐challenged mice or OVA‐sensitized and OVA‐challenged mice, with or without clopidogrel treatment. Then, β‐actin was used as a loading control.*P <0.05 versus OVA/OVA group.

To determine the expression of P2Y12, the signals of P2Y12 were detected through western blot after depleting platelets with antiplatelet antibody. The expression of P2Y12 was increased markedly following OVA challenge in OVA‐sensitized mice compared to OVA‐sensitized and saline‐challenged mice, and this expression was not attenuated by clopidogrel administration in OVA‐sensitized and ‐challenged mice (Fig. 1).

### Clopidogrel treatment prevents the development of AHR and airway inflammation

**Figure 2:**
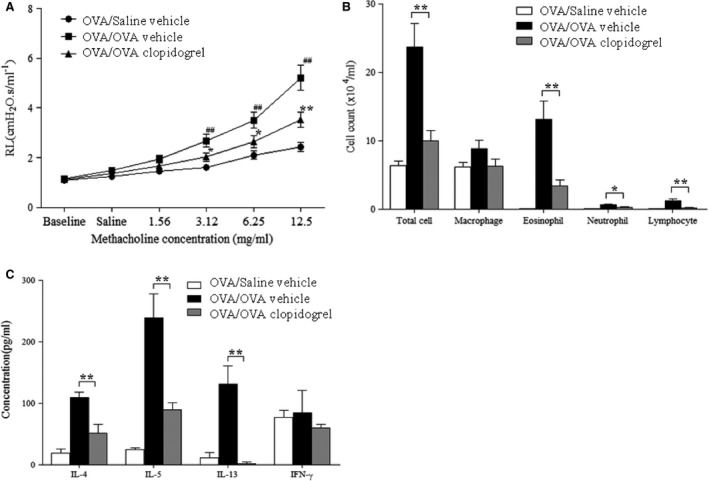
The effect of clopidogrel treatment on airway responses in an OVA‐specific asthma model. (**A**) Changes in lung resistance by increasing methacholine 0, 1.56, 3.12, 6.25 and 12.5 mg/kg were assessed 48 hrs after the final challenge. (**B**) Cell composition in BAL fluid. (**C**) The levels of IL‐4, IL‐5, IL‐13 and IFN‐γ were determined by using ELISA in BAL fluid. OVA/Saline vehicle, mice sensitized with OVA and challenged with saline; OVA/OVA vehicle, mice sensitized and challenged with OVA; OVA/OVA clopidogrel treatment, OVA challenged mice treated with clopidogrel. The data are expressed as the mean ± SEM. *n* = 15 for each group. **P < 0.01 and *P < 0.05 *versus* the OVA/OVA vehicle group. ##P <0 .01 versus the OVA/saline vehicle group. RL, resistance of lung; OVA, ovalbumin; IL, interleukin; IFN, interferon.

To find out the blocking effects of clopidogrel on allergen‐induced AHR and airway inflammation, mice were treated with 30 mg/kg of clopidogrel during the OVA‐challenge phase. Following OVA sensitisation and challenge, vehicle‐treated mice had significantly a higher lung resistance responding to MCh as well as a larger numbers of inflammatory cells, including eosinophils in BAL fluid compared to OVA‐sensitized and saline‐challenged mice (*P* < 0.01). Clopidogrel treated mice developed significantly a lower airway responsiveness to inhaled MCh and a smaller number of eosinophils in BAL fluid compared to vehicle‐treated mice (*P* < 0.01) (Fig. 2A and B). As shown in Figure 2C, clopidogrel treatment reduced the levels of IL‐4, IL‐5 and IL‐13 in BAL fluid, but we did not find a significant difference in the IFN‐γ level compared to vehicle‐treated mice.

**Figure 3:**
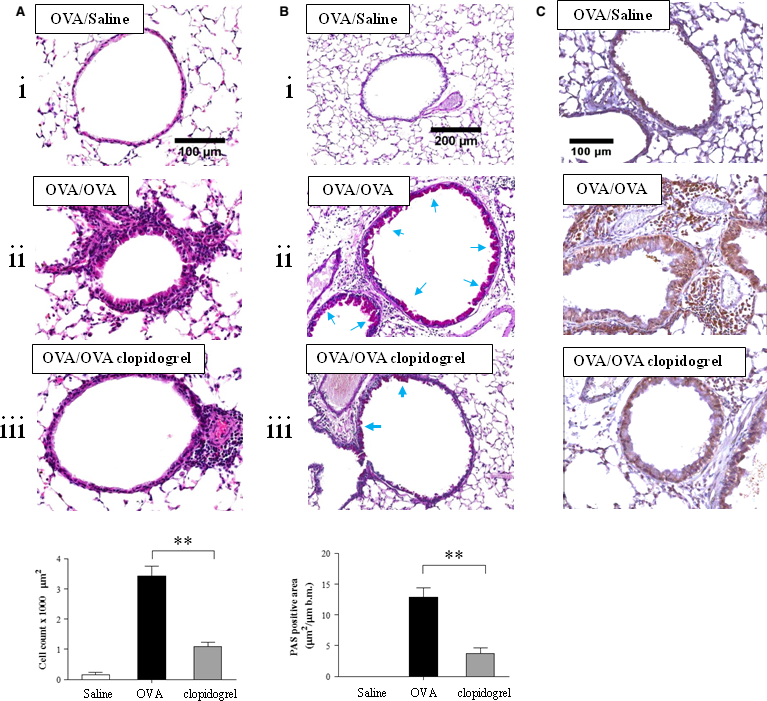
The effect of clopidogrel treatment in lung tissue. (**A**) Lung tissue histology with haematoxylin and eosin and (**B**) periodic acid‐Schiff (PAS) staining. Quantitative analysis of inflammatory and PAS ^+^ cells in lung tissue was performed as described in the Materials and Methods section. (**C**) Immunohistochemistry of ECP was performed with an anti‐mouse ECP antibody. (i) Mice sensitized with OVA and challenged with saline (OVA/Saline vehicle); (ii) mice sensitized and challenged with OVA (OVA/OVA vehicle); (iii) OVA challenged mice treated with clopidogrel (OVA/OVA clopidogrel treatment). **P < 0.01 *versus* the OVA/OVA group.

Histopathological examination of lung tissue sections revealed that the numbers of inflammatory cells, including eosinophils, in peribronchial and perivascular areas were increased in OVA‐sensitized and ‐challenged mice. Similarly, the numbers of PAS‐positive mucus‐containing goblet cells were also increased in the same mice. However, administration of clopidogrel significantly decreased the numbers of inflammatory cells and PAS‐positive mucus‐containing goblet cells in lung tissue (Fig. 3A and B). As shown in Figure 3C, the expression of ECP in peribronchial and perivascular areas was increased in OVA‐sensitized and ‐challenged mice compared to OVA ‐sensitized and saline‐challenged mice. However, clopidogrel treatment significantly decreased the expression of ECP in lung tissue.

### Eosinophils present P2Y12 receptors, and clopidogrel treatment decreases the secretion of chemokine CC ligand 5 in lung tissue

**Figure 4:**
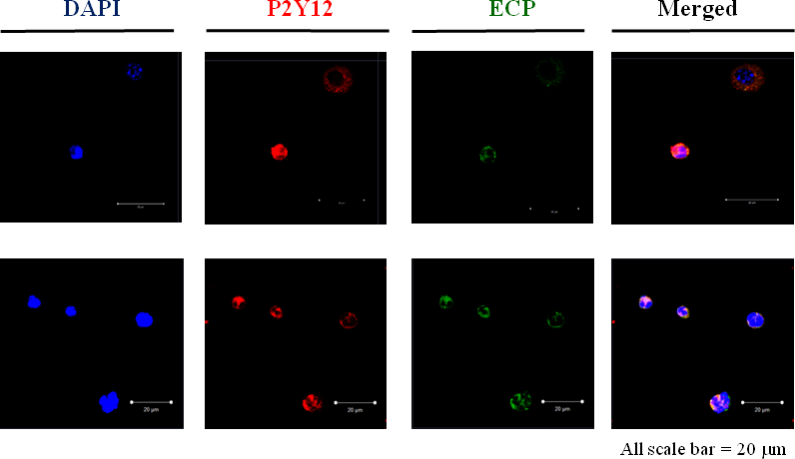
The presence of the P2Y12 receptor in eosinophils in BAL fluid. Detection of P2Y12 in BAL cells from a representative OVA‐sensitized and ‐challenged mouse. Cells were incubated with anti‐P2Y12 receptor or anti‐ECP antibodies. Upper and lower panels are two representative photographs in each group. Analysis was performed by confocal microscopy.

Next, the presence of P2Y12 receptors on eosinophils was investigated. Double immunocytochemistry with anti‐ECP and anti‐P2Y12 antibodies revealed that P2Y12 was co‐localized to eosinophils in BAL fluid (Fig. 4).

**Figure 5:**
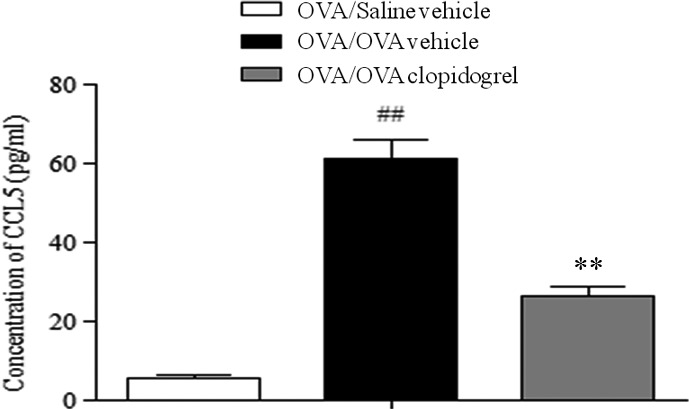
The effect of clopidogrel treatment on the secretion of CCL5 in BAL fluid. The levels of CCL5 were determined by using ELISA in BAL fluid. ##*P* < 0.01 *versus* the OVA/Saline vehicle group. **P < 0.01 *versus* the OVA/OVA vehicle group. BAL, bronchial alveolar lavage; CCL5, chemokine CC ligand 5.

To investigate how clopidogrel could attenuate airway eosinophilic inflammation, CCL5, an eosinophilic chemoattractant, in BAL fluid, the expressions of adherent molecules in lung homogenates were measured in clopidogrel‐treated and vehicle‐treated mice. The level of CCL5 in BAL fluid was significantly decreased in clopidogrel‐treated mice compared to vehicle‐treated mice (Fig. 5). However, clopidogrel treatment did not affect the expression of VCAM‐1 or ICAM‐1 in lung homogenates (data not shown).

### Clopidogrel increases intracellular expression of ECP in eosinophils and decreases its extracellular expression after LTE4 stimulation

**Figure 6:**
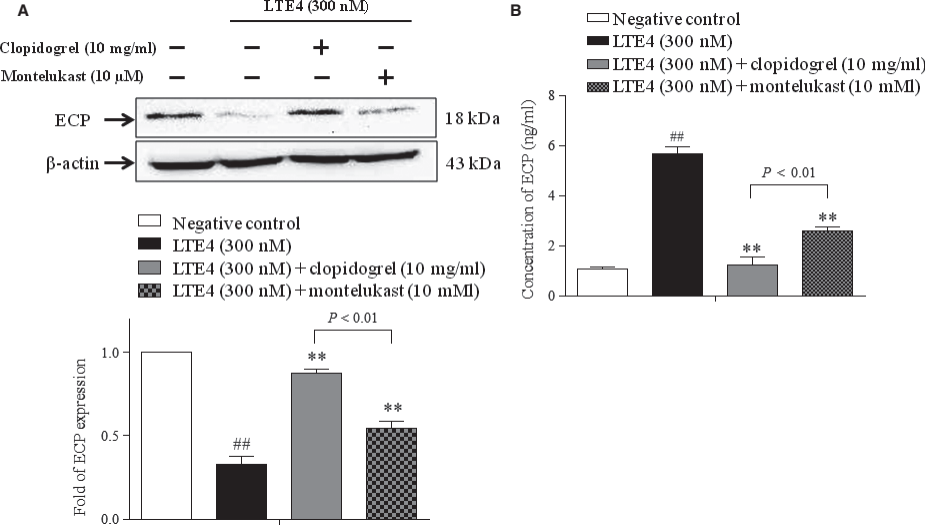
The effects of clopidogrel and montelukast on ECP expression in human eosinophils. Cells were pre‐treated with clopidogrel (10 μg/ml) or montelukast (10 μM) in serum‐free media for 30 min., and then were stimulated with 300 nM of LTE4 for further 30 min. (**A**) The intracellular expression of ECP in EOL‐1 cell investigated by using Western blot. (**B**) The level of secreted ECP in cell culture supernatants evaluated by using ELISA. ##*P* < 0.01 compared to the negative control group; **P < 0.01 compared to the LTE4‐treated group. *P*‐values were obtained by using one‐way anova.

Finally, intracellular and extracellular expressions of ECP were measured using EOL‐1 cells that were stimulated with LTE4, with or without clopidogrel. The intracellular level of ECP was decreased by LTE4 stimulation without clopidogrel; however, clopidogrel treatment significantly inhibited the decrease in the intracellular ECP level induced by LTE4 stimulation. These results were more significantly observed in the clopidogrel‐treated group than in the montelukast‐treated group (*P* < 0.01, Fig. 6A). In contrast, as shown in Figure 6B, the levels of ECP in the supernatant were significantly increased by LTE4 stimulation without clopidogrel (*P* < 0.01); both clopidogrel and montelukast treatments inhibited the increase in the ECP level induced by LTE4 stimulation (*P* < 0.01 for each). Notably, the inhibiting effect in the supernatant was significantly greater in the clopidogrel group than in the montelukast group (*P* < 0.01). Taken together, clopidogrel inhibited extracellular ECP secretion induced by LTE4 stimulation.

## Discussion

Asthma is characterized by increased infiltration of inflammatory cells, including eosinophils, AHR, reversible airway obstruction along with mucus hypersecretion, and lung remodeling enhanced by cysLTs, such as LTC4, LTD4 and LTE4 22, 23. There are at least 3G‐protein‐coupled receptors for cysLTs, including CysLT1R, CysLT2R 16, and newly investigated CysLT3R 14, 24. LTD4 and LTC4 strongly bind to CysLT1R and CysLT2R, respectively, which are found to be expressed by various cells of the immune system 22. Montelukast, is a well‐known CysLTR1R antagonist that competes with LTD4 to bind its receptor, and thereby attenuate bronchoconstriction and airway inflammation induced by CysLTs 25. However, the last and most stable metabolite of CysLT is LTE4, which has been shown to be highly increased in the urine of asthma patients compared to healthy individuals 26. Nasal administration of LTE4 increases infiltration and accumulation of various inflammatory cells in the murine lung tissue 24. LTE4 has been found to bind weakly to CysLT1R as well as CysLT2R; therefore, many investigators have attempted to identify high‐affinity receptors for LTE4. Recently, several novel candidate receptors for LTE4, including GPR 99 and P2Y12R, have been suggested 7, 14, 15. First, P2Y12R is predicted to be the third CysLTR through *in silico* analysis and *in vitro* investigation which showed that LTE4 elicits calcium flux and that phosphorylation of extracellular signals regulats kinase levels in P2Y12R‐overexpressing transfected cells in a dependent manner 13. In addition, Paruchuri *et al*. 24 have shown that P2Y12R is required in LTE4‐mediated pulmonary eosinophilic inflammation. However, a more recent study has demonstrated that LTE4 does not activate signalling through P2Y12R because no calcium mobilization is elicited in P2Y12‐expressing cells 14. Instead, GPR 99 has been identified as a new candidate receptor for LTE4 15, 27. Therefore, P2Y12R is simply regarded as a co‐receptor for LTE4 28.

Several studies have reported the inhibitory effect of P2Y12 receptor antagonists on inflammatory responses 24, 29. We found that the expression of P2Y12 was increased after allergen challenge in allergen‐sensitized mice. Next, we evaluated the protective role of clopidogrel, a P2Y12 receptor antagonist, in the pathogenesis of asthma by using a mouse model of asthma and *in vitro* experiments. A previous study has reported that mucus secretion and inflammatory cell infiltration into the airways of asthmatic mice are decreased in P2Y12^−/−^ and clopidogrel‐treated mice 24. This is consistent with our result that administration of clopidogrel decreased the numbers of airway inflammatory cells, including eosinophils, in BAL fluid as well as lung tissue. In addition, we first provided the evidence to indicate that clopidogrel treatment decreases AHR to Mch and secretion of Th2 cytokines in mouse BAL fluid.

Eosinophil is the most important effector cell, and eosinophilia is one of the characteristics of allergic inflammation 30. Eosinophils infiltrating into the airway undergo primary lysis and release free eosinophil granules containing most of the important mediators, including ECP. Additionally, the mediators released from eosinophil granules are known to play a crucial role in tissue remodeling in patients with asthma 31. In this study, attenuation of eosinophilia after treatment with clopidogrel showed a striking feature in BAL fluid compared to other studies that used montelukast 32. This finding suggested that clopidogrel may have a major effect on eosinophil activation and function.

It has been known that LTE4 stimulation increases the infiltration of eosinophils into the lungs in asthma patients and releases ECP from isolated human eosinophils 4, 24. Therefore, we further investigated the influence of clopidogrel treatment on the activation of eosinophils. As expected, we found that LTE4 stimulation decreases intracellular expression of ECP and increases its extracellular secretion from human eosinophils, which indicates that LTE4 could trigger degranulation of eosinophils. In contrast, clopidogrel treatment inhibited the decrease in intracellular ECP expression induced by LTE4 stimulation. Similar results were obtained in the lung tissue from OVA‐sensitized and ‐challenged mice treated with clopidogrel. These findings suggest that clopidogrel can inhibit LTE4‐mediated activation of eosinophils *in vivo* and *in vitro*.

However, clopidogrel may have an impact on other inflammatory cells such as T cells and platelets. CCL5 which was downregulated by clopidogrel is mainly secreted from T cell, and the depletion of inhibition of recruitment of eosinophils did not change cytokines in BAL fluid 33, 34, 35. Instead, the function of T cells seems to be altered by the treatment of clopidogrel. T cells can reduce the expression of Th2 cytokine, mucus production and eosinophil number in the airway through reduction of CCL5. However, T cells do not express P2Y12. Therefore, the effects of clopidogrel on T cells could be indirect. Other cell types such as platelets may play a role in suppression of allergic airway dysfunction and inflammation following clopidogrel treatment. There is a possibility that platelet‐eosinophil complex in lung tissue and BAL cell have a role in the effect of clopidogrel in our asthma model. The binding of activated platelets to eosinophils by P‐selectin occur in both non‐severe and severe asthma and this complex may affect the activation of eosinophil 16. Adhesion molecules are known to mediate migration of intravascular immune cells to surrounding tissues during inflammatory responses 36, 37. In this study, we found the increased expression of VCAM and ICAM in lung tissue homogenates obtained from OVA‐sensitized and ‐challenged mice, which were not inhibited by clopidogrel treatment. Based on these results, it is conceivable that the inhibitory effect of clopidogrel could be mediated by production and/or secretion of several chemokines, but not inhibition of inflammatory cell migration.

Recently, Lussana *et al*. published the first, randomized, double‐blind, placebo‐controlled study that pharmacologic inhibition of P2Y12 receptor may be useful in the treatment of asthma using prasugrel, a third generation P2Y12 inhibitor. They showed that significantly improved AHR after taking 10 mg of prasugrel for 15 days in 26 asthma patients 38. Further studies are needed to confirm the effect of P2Y12 inhibition in asthma.

In conclusion, our study provides clear evidence to indicate that clopidogrel, a P2RY12 antagonist, attenuates allergic inflammation and AHR in asthma. The results of this study suggest that P2Y12 antagonists can be used as novel therapeutics for asthma.

## Conflicts of interest

The authors confirm that there are no conflicts of interest.

## References

[1] Blanchard C , Rothenberg ME . Biology of the eosinophil. Adv Immunol. 2009; 101: 81–121. 19231593 10.1016/S0065-2776(08)01003-1 PMC4109275

[2] Rothenberg ME , Hogan SP . The eosinophil. Annu Rev Immunol. 2006; 24: 147–74. 16551246 10.1146/annurev.immunol.24.021605.090720

[3] Gleich GJ . Mechanisms of eosinophil‐associated inflammation. J Allergy Clin Immunol. 2000; 105: 651–63. 10756213 10.1067/mai.2000.105712

[4] Neves JS , Radke AL , Weller PF . Cysteinyl leukotrienes acting *via* granule membrane‐expressed receptors elicit secretion from within cell‐free human eosinophil granules. J Allergy Clin Immunol. 2010; 125: 477–82. 20159258 10.1016/j.jaci.2009.11.029 PMC2824614

[5] Peters‐Golden M , Henderson WR Jr . Leukotrienes. N Engl J Med. 2007; 357: 1841–54. 17978293 10.1056/NEJMra071371

[6] Sala A , Voelkel N , Maclouf J , *et al* Leukotriene E4 elimination and metabolism in normal human subjects. J Biol Chem. 1990; 265: 21771–8. 2174886

[7] Laitinen LA , Laitinen A , Haahtela T , *et al* Leukotriene E4 and granulocytic infiltration into asthmatic airways. Lancet. 1993; 341: 989–90. 8096945 10.1016/0140-6736(93)91073-u

[8] Gauvreau GM , Parameswaran KN , Watson RM , *et al* Inhaled leukotriene E(4), but not leukotriene D(4), increased airway inflammatory cells in subjects with atopic asthma. Am J Respir Crit Care Med. 2001; 164: 1495–500. 11704602 10.1164/ajrccm.164.8.2102033

[9] Lynch KR , O'Neill GP , Liu Q , *et al* Characterization of the human cysteinyl leukotriene CysLT1 receptor. Nature. 1999; 399: 789–93. 10391245 10.1038/21658

[10] Heise CE , O'Dowd BF , Figueroa DJ , *et al* Characterization of the human cysteinyl leukotriene 2 receptor. J Biol Chem. 2000; 275: 30531–6. 10851239 10.1074/jbc.M003490200

[11] Woszczek G , Chen LY , Nagineni S , *et al* IFN‐gamma induces cysteinyl leukotriene receptor 2 expression and enhances the responsiveness of human endothelial cells to cysteinyl leukotrienes. J Immunol. 2007; 178: 5262–70. 17404310 10.4049/jimmunol.178.8.5262

[12] Woszczek G , Chen LY , Nagineni S , *et al* Leukotriene D(4) induces gene expression in human monocytes through cysteinyl leukotriene type I receptor. J Allergy Clin Immunol. 2008; 121: 215–21 e1. 18028998 10.1016/j.jaci.2007.09.013 PMC5560436

[13] Nonaka Y , Hiramoto T , Fujita N . Identification of endogenous surrogate ligands for human P2Y12 receptors by *in silico* and *in vitro* methods. Biochem Biophys Res Commun. 2005; 337: 281–8. 16185654 10.1016/j.bbrc.2005.09.052

[14] Foster HR , Fuerst E , Lee TH , *et al* Characterisation of P2Y(12) receptor responsiveness to cysteinyl leukotrienes. PLoS One. 2013; 8: e58305. 23472176 10.1371/journal.pone.0058305 PMC3589271

[15] Kanaoka Y , Maekawa A , Austen KF . Identification of GPR99 protein as a potential third cysteinyl leukotriene receptor with a preference for leukotriene E4 ligand. J Biol Chem. 2013; 288: 10967–72. 23504326 10.1074/jbc.C113.453704 PMC3630866

[16] Laidlaw TM , Kidder MS , Bhattacharyya N , *et al* Cysteinyl leukotriene overproduction in aspirin‐exacerbated respiratory disease is driven by platelet‐adherent leukocytes. Blood. 2012; 119: 3790–8. 22262771 10.1182/blood-2011-10-384826 PMC3335383

[17] Losol P , Palikhe NS , Lee JW , *et al* Association of P2RY12 polymorphisms with eosinophil and platelet activation in patients with aspirin‐exacerbated respiratory disease. Ann Allergy Asthma Immunol. 2015; 114: 423–4 e1 25778862 10.1016/j.anai.2015.02.009

[18] Foster CJ , Prosser DM , Agans JM , *et al* Molecular identification and characterization of the platelet ADP receptor targeted by thienopyridine antithrombotic drugs. J Clin Invest. 2001; 107: 1591–8. 11413167 10.1172/JCI12242 PMC200194

[19] Takeda K , Miyahara N , Kodama T , *et al* S‐carboxymethylcysteine normalises airway responsiveness in sensitised and challenged mice. Eur Respir J. 2005; 26: 577–85. 16204586 10.1183/09031936.05.00090304

[20] Tarkowski M , Vanoirbeek JA , Vanhooren HM , *et al* Immunological determinants of ventilatory changes induced in mice by dermal sensitization and respiratory challenge with toluene diisocyanate. Am J Physiol Lung Cell Mol Physiol. 2007; 292: L207–14. 16963530 10.1152/ajplung.00157.2005

[21] Tomkinson A , Cieslewicz G , Duez C , *et al* Temporal association between airway hyperresponsiveness and airway eosinophilia in ovalbumin‐sensitized mice. Am J Respir Crit Care Med. 2001; 163: 721–30. 11254531 10.1164/ajrccm.163.3.2005010

[22] Kanaoka Y , Boyce JA . Cysteinyl leukotrienes and their receptors: cellular distribution and function in immune and inflammatory responses. J Immunol. 2004; 173: 1503–10. 15265876 10.4049/jimmunol.173.3.1503

[23] Barnes PJ , Chung KF , Page CP . Inflammatory mediators of asthma: an update. Pharmacol Rev. 1998; 50: 515–96. 9860804

[24] Paruchuri S , Tashimo H , Feng C , *et al* Leukotriene E4‐induced pulmonary inflammation is mediated by the P2Y12 receptor. J Exp Med. 2009; 206: 2543–55. 19822647 10.1084/jem.20091240 PMC2768854

[25] Takeda K , Shiraishi Y , Matsubara S , *et al* Effects of combination therapy with montelukast and carbocysteine in allergen‐induced airway hyperresponsiveness and airway inflammation. Br J Pharmacol. 2010; 160: 1399–407. 20590630 10.1111/j.1476-5381.2010.00797.x PMC2938811

[26] Sampson AP , Castling DP , Green CP , *et al* Persistent increase in plasma and urinary leukotrienes after acute asthma. Arch Dis Child. 1995; 73: 221–5. 7492159 10.1136/adc.73.3.221 PMC1511288

[27] Kanaoka Y , Boyce JA . Cysteinyl leukotrienes and their receptors; emerging concepts. Allergy Asthma Immunol Res. 2014; 6: 288–95. 24991451 10.4168/aair.2014.6.4.288 PMC4077954

[28] Steinke JW , Negri J , Payne SC , *et al* Biological effects of leukotriene E4 on eosinophils. Prostaglandins Leukot Essent Fatty Acids. 2014; 91: 105–10. 24768603 10.1016/j.plefa.2014.02.006 PMC4127125

[29] Garcia AE , Mada SR , Rico MC , *et al* Clopidogrel, a P2Y12 receptor antagonist, potentiates the inflammatory response in a rat model of peptidoglycan polysaccharide‐induced arthritis. PLoS One. 2011; 6: e26035. 22028806 10.1371/journal.pone.0026035 PMC3196585

[30] Fahy JV . Eosinophilic and neutrophilic inflammation in asthma: insights from clinical studies. Proc Am Thorac Soc. 2009; 6: 256–9. 19387026 10.1513/pats.200808-087RM

[31] Persson C . Primary lysis of eosinophils in severe desquamative asthma. Clin Exp Allergy. 2014; 44: 173–83. 24330324 10.1111/cea.12255

[32] Wu AY , Chik SC , Chan AW , *et al* Anti‐inflammatory effects of high‐dose montelukast in an animal model of acute asthma. Clin Exp Allergy. 2003; 33: 359–66. 12614451 10.1046/j.1365-2222.2003.01615.x

[33] Das AM , Vaddi KG , Solomon KA , *et al* Selective inhibition of eosinophil influx into the lung by small molecule CC chemokine receptor 3 antagonists in mouse models of allergic inflammation. J Pharmacol Exp Ther. 2006; 318: 411–7. 16614169 10.1124/jpet.105.099812

[34] Wegmann M , Goggel R , Sel S , *et al* Effects of a low‐molecular‐weight CCR‐3 antagonist on chronic experimental asthma. Am J Respir Cell Mol Biol. 2007; 36: 61–7. 16917075 10.1165/rcmb.2006-0188OC

[35] Jacobsen EA , Lesuer WE , Willetts L , *et al* Eosinophil activities modulate the immune/inflammatory character of allergic respiratory responses in mice. Allergy. 2014; 69: 315–27. 24266710 10.1111/all.12321 PMC3944108

[36] Stanciu LA , Djukanovic R . The role of ICAM‐1 on T‐cells in the pathogenesis of asthma. Eur Respir J. 1998; 11: 949–57. 9623703 10.1183/09031936.98.11040949

[37] Liou CJ , Cheng PY , Huang WC , *et al* Oral lovastatin attenuates airway inflammation and mucus secretion in ovalbumin‐induced murine model of asthma. Allergy Asthma Immunol Res. 2014; 6: 548–57. 25374755 10.4168/aair.2014.6.6.548 PMC4214976

[38] Lussana F , Di Marco F , Terraneo S , *et al* Effect of prasugrel in patients with asthma: results of PRINA, a randomized, double‐blind, placebo‐controlled, cross‐over study. J Thromb Haemost. 2015; 13: 136–41. 25387888 10.1111/jth.12779

